# Effects of Angiotensin II Receptor 1 Inhibition by LCZ696 on the Acquisition and Relapse of Methamphetamine-Associated Contextual Memory

**DOI:** 10.3390/ph18071016

**Published:** 2025-07-08

**Authors:** Xiaofang Li, Zhiting Zou, Xiangdong Yang, Jinnan Lü, Xiaoyu Zhang, Jiahui Zhou, Dan Zhu, Xinshuang Gong, Shujun Lin, Zhaoying Yu, Zizhen Si, Wenting Wei, Yakai Xie, Yu Liu

**Affiliations:** 1 Department of Psychology, College of Teacher Education, Ningbo University, Ningbo 315211, China; 2 School of Pharmacy, Health Science Center, Ningbo University, Ningbo 315211, China; 3 School of Basic Medical Sciences, Health Science Center, Ningbo University, Ningbo 315021, China; 4 School of Public Health, Health Science Center, Ningbo University, Ningbo 315021, China; 5 Affiliated Kangning Hospital, Ningbo University, Ningbo 315211, China; 6 School of Materials Science and Chemical Engineering, Ningbo University, Ningbo 315211, China; 7 Health BioMed Co., Ltd., Ningbo 315040, China

**Keywords:** methamphetamine, rewarding effect, LCZ696, angiotensin II type I receptor II I, Creb

## Abstract

**Background/Objectives:** Contextual memory associated with methamphetamine (METH) use contributes to relapse and persistence of addiction. Angiotensin II type 1 receptor (AT1R) signaling has been implicated in drug reinforcement. LCZ696, a clinically used combination of sacubitril (a neprilysin inhibitor) and valsartan (an AT1R antagonist), may interfere with METH-associated memory through the modulation of dopaminergic pathways. **Methods:** Male C57BL/6J mice were tested in a conditioned place preference (CPP) paradigm to assess the effects of LCZ696, sacubitril (AHU377), and valsartan on METH-induced memory expression and reinstatement. Synaptic plasticity in the nucleus accumbens (NAc) was examined by assessing the levels of synaptophysin (Syp) and postsynaptic density protein 95 (Psd95), as well as dendritic spine density. Dopaminergic signaling in the ventral tegmental area (VTA) was evaluated via ELISA, Western blotting, and chromatin immunoprecipitation (ChIP), targeting cAMP response element-binding protein (Creb) binding to the tyrosine hydroxylase (*Th*) promoter. To further assess the role of Th, an adeno-associated virus (AAV9) carrying a CRISPR-Cas9-based sgRNA targeting Th (AAV9-Th-sgRNA) was microinjected into the VTA. **Results:** LCZ696 and valsartan significantly reduced METH-induced CPP and reinstatement. LCZ696 reversed METH-induced synaptic and dopaminergic alterations and suppressed Creb-mediated *Th* transcription. *Th* knockdown attenuated both CPP acquisition and relapse. **Conclusions**: LCZ696 disrupts METH-associated contextual memory by modulating dopaminergic signaling and Creb-dependent Th expression, supporting its potential as a treatment for METH use disorder.

## 1. Introduction

Methamphetamine (METH) use disorder is a chronic, relapsing neuropsychiatric condition characterized by the interplay of the drug reward and cognitive impairment [1,2]. Repeated METH exposure induces dysfunction across multiple brain regions involved in reward processing, such as the nucleus accumbens (NAc) and ventral tegmental area (VTA), primarily through dysregulation of the dopaminergic system, other neurotransmitter systems, including glutamatergic, GABAergic, and serotonergic pathways [3,4,5]. Cognitive impairments associated with METH use are linked to impairments in the prefrontal cortex and hippocampus, and these widespread neuroadaptations are further exacerbated by chronic neuroinflammation, oxidative stress, and neurotransmitter systems [6,7,8,9,10]. Up to date, there is still no effective pharmaceutical treatment for METH use disorder. Growing evidence suggests that the brain possesses an intrinsic renin–angiotensin system (RAS), which involves angiotensin II (Ang II), angiotensin-converting enzyme (ACE), angiotensin II type I receptor (AT1R), and angiotensin II type II receptor (AT2R) [11]. While angiotensin is considered a minor component among the major neurotransmitter systems in the brain, the central RAS may still modulate specific neurological processes, primarily via AT1R activation. Clinically, ACE inhibitors were initially explored to manage cardiovascular symptoms in amphetamine users, with perindopril showing a complex modulation of methamphetamine-induced hemodynamic and subjective effects, though mechanistic insights remained limited [12]. For example, patients with Alzheimer’s disease exhibit increased neuroinflammation, oxidative stress, tau pathology, and amyloid-β deposition—key pathogenic mechanisms that are accompanied by elevated AT1R expression [13]. The AT1R antagonist, losartan, has demonstrated potential in alleviating anxiety-like behaviors, pain perception, and cognitive impairment induced by stress [14]. Moreover, studies modulating AT1R and AT2R have demonstrated promising results in neuroprotection against ischemic brain injuries [15].

Studies have revealed that Ang II was involved in the regulation of the central dopamine (DA) system [11]. For instance, Ang II has been shown to influence DA levels by reducing AT1R expression in the substantia nigra of patients with Parkinson’s disease [16]. Moreover, losartan has also been found to reduce Ang II-induced striatal DA release [11]. Another study has demonstrated that the ACE inhibition by captopril enhanced endogenous opioid signaling [17]. Chronic METH use also been shown to result in increased AT1R mRNA and protein expression in the striatum [18]. For example, patients with Alzheimer’s disease exhibit increased neuroinflammation, oxidative stress, tau pathology, and amyloid-β deposition—key pathogenic mechanisms that are accompanied by elevated AT1R expression. Furthermore, the inhibition of AT1R by candesartan cilexetil significantly decreased METH self-administration (SA) and cue-/drug-induced reinstatement in rats via the phospholipase C β1 (PLCβ1)–cAMP response element-binding protein (Creb) signaling pathway [18,19]. The AT1R inhibition by losartan has also been demonstrated to reduce the impact of negative outcomes during learning, showing promising therapeutic potential to normalize distorted reward learning in depression [19]. These results indicate that the modulation of Ang II and the receptors could be a new direction for the development of pharmaceutical agents to treat METH use disorder. In line with this, a recent study demonstrated that sacubitril/valsartan (LCZ696) improved cognitive impairments induced by methamphetamine exposure, further supporting its therapeutic potential in METH-related neuropsychiatric conditions [20].

LCZ696 is a newly developed combination drug that comprises valsartan and sacubitril (AHU377). It produces dual effects that both block AT1R and neprilysin (NEP). NEP is a zinc-dependent metalloprotease involved in the degradation of several neuropeptides, such as enkephalins, which modulate pain and reward. NEP inhibition may influence dopaminergic and opioid signaling pathways, which are implicated in drug abuse disorders [21].

This study was designed to investigate the effects of LCZ696 on METH-induced conditioned place preference (CPP), a behavioral paradigm reflecting drug-associated contextual reward, and to elucidate the underlying molecular mechanisms. We first evaluated the impact of LCZ696 on both the acquisition and reinstatement of METH-induced CPP. At the molecular level, phosphorylated cAMP response element-binding protein (Creb) binds to the cAMP response element (CRE) within the promoter region of the *Th* gene, thereby regulating its transcription. Th is the rate-limiting enzyme for DA synthesis. To explore whether LCZ696 modulates Creb-driven Th transcription and DA production, we employed a viral-mediated gene manipulation approach targeting the VTA. Collectively, these findings suggest that targeting angiotensin II signaling—particularly through AT1R inhibition—may provide a novel therapeutic strategy for disrupting drug-associated contextual memory and mitigating relapses in METH use disorder.

## 2. Results

### 2.1. Experiment 1: The Effect of LCZ696, AHU337, and Valsartan on METH CPP

**Figure 1:**
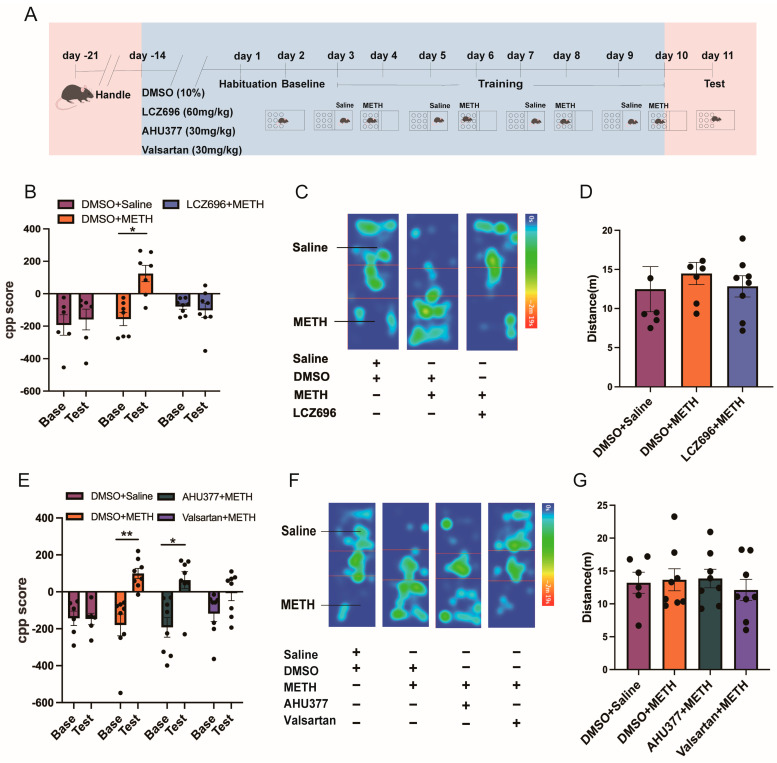
The effect of LCZ696, AHU337, and valsartan on METH-associated contextual memory acquisition. (**A**) The experimental timeline. (**B**) LCZ696 decreased CPP score in mice exposed to METH. (**C**) Heat map of the mice’s trajectory in the CPP apparatus. The color of the bar from 0 s to 2 m 19 s on the right side of the panel changes from blue to red, which represents the time the mouse spends in one area. (**D**) The locomotor distance of the spontaneous activity test in mice administered with LCZ696. (**E**) The effect of valsartan and AHU377 on CPP score in mice exposed to METH. (**F**) Heat map of the mice’s trajectory in the CPP apparatus. The color of the bar from 0 s to 2 m 19 s on the right side of the panel changes from blue to red, which represents the time the mouse spends in one area. (**G**) The locomotor distance of the spontaneous activity test in mice administered with AHU377 and valsartan. * *p* < 0.05, ** *p* < 0.01.

The Scheirer–Ray–Hare test showed that among the DMSO + saline, DMSO + METH, and LCZ696 + METH groups, the interaction effect (H_(2,36)_ = 6.245, *p* < 0.05) had a significant impact on CPP scores. However, the main effects of testing (H_(1,36)_ = 3.802, *p* > 0.05) and treatment (H_(2,36)_ = 3.931, *p* > 0.05) were not significant. Further post hoc comparisons revealed no significant difference between the CPP baseline (Base) and test scores in the DMSO + saline and LCZ696 + saline groups (*p* > 0.05). In contrast, the CPP Base score in the DMSO + METH group was significantly higher than the test score (*p* < 0.05; Figure 1B,C). There was no significant difference in locomotor activity among the groups (H_(2,18)_ = 0.3181, *p* > 0.05; Figure 1D).

The Scheirer–Ray–Hare test indicated that among the DMSO + saline, DMSO + METH, AHU377 + METH, and valsartan + METH groups, testing had a significant effect on CPP scores (H_(1,52)_ = 17.45, *p* < 0.001), while treatment (H_(3,52)_ = 5.36, *p* > 0.05) and the test × treatment interaction (H_(3,52)_ = 7.63, *p* > 0.05) were not significant. Further post hoc analysis showed no significant difference between the CPP Base and test scores in the DMSO + saline and valsartan + METH groups (*p* > 0.05). In contrast, the CPP Base scores in the DMSO + METH (*p* < 0.01) and AHU377 + METH (*p* < 0.05) groups were significantly higher than their test scores (Figure 1E,F). There was no significant difference in locomotor activity among the groups (H_(3,26)_ = 0.2572, *p* > 0.05; Figure 1G).

### 2.2. Experiment 2: The Effect of LCZ696, AHU337, and Valsartan on METH CPP Retrieval

**Figure 2:**
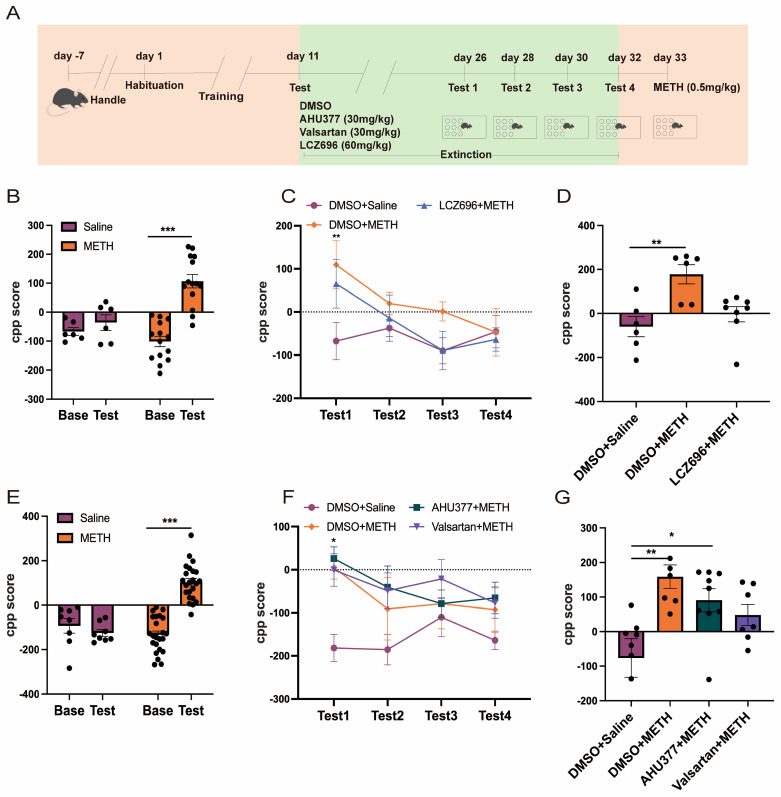
The effect of LCZ696, HUA377, and valsartan on METH-induced robust relapse in METH CPP acquisition mice. (**A**) The experimental timeline. (**B**) CPP score of METH-associated contextual memory acquisition mice. (**C**) CPP score of retrieval in the DMSO + saline, DMSO + METH, and LCZ696 + METH groups. (**D**) CPP score of METH-induced robust relapse in METH CPP acquisition mice. (**E**) CPP score of METH-associated contextual memory acquisition mice. (**F**) CPP score of retrieval in the DMSO + saline, DMSO + METH, AHU377 + METH, and valsartan + METH groups. (**G**) CPP score of METH-induced robust relapse in METH CPP acquisition mice. * *p* < 0.05, ** *p* < 0.01, *** *p* < 0.001.

Two-way repeated measures ANOVA showed that there was a major effect of the test, treatment, and test × treatment interaction on the CPP scores between the saline and METH groups (F_(1,36)_ = 23.72, *p* < 0.001; F_(1,36)_ = 4.837, *p* < 0.05; F_(1,36)_ = 13.01, *p* < 0.001). Post hoc analysis showed that there was no significant difference between the CPP base and test scores in the saline group (*p* > 0.05). The METH group exhibited significantly higher CPP test scores than Base scores (*p* < 0.001; Figure 2B). Two-way repeated measures ANOVA revealed a major effect of test (F_(3,68)_ = 3.041, *p* < 0.05) and treatment (F_(2,68)_ = 3.189, *p* < 0.05) on the CPP scores among the DMSO + saline, DMSO + METH, and LCZ696 + METH groups during the phase of extinction. There was no significant effect of the test × treatment interaction (F_(6,68)_ = 0.9382, *p* > 0.05). Further post hoc analysis showed both the DMSO + METH and LCZ6969 + METH groups produced significantly higher CPP scores than the DMSO + saline group during Test 1 (*p* < 0.01). There were no significant differences in the CPP scores between the DMSO + saline and DMSO + METH groups during Test 2, Test 3, and Test 4 (*p* > 0.05 Figure 2C). The Kruskal–Wallis test indicated that during the retrieval phase, treatment had a statistically significant effect on CPP scores among the DMSO + saline, DMSO + METH, and LCZ696 + METH groups (H = 12.56, *p* < 0.01). Post hoc analysis showed that the DMSO + METH group exhibited significantly higher CPP scores compared to the DMSO + saline group (*p* < 0.01). However, there was no significant difference in CPP scores between the DMSO + METH and LCZ696 + METH groups (*p* > 0.05; Figure 2D).

Two-way repeated measures ANOVA demonstrated significant effects of the test, treatment, and test × treatment interaction on the CPP scores between saline and METH groups (F_(1,58)_ = 19.94, *p* < 0.001; F_(1,58)_ = 17.01, *p* < 0.001; F_(1,58)_ = 34.43, *p* < 0.001). The post hoc analysis indicated that there were no significant differences between the CPP Base and test scores in the saline group (Figure 2E; *p* > 0.05). The CPP test was significantly higher than the baseline scores in the METH group (*p* < 0.001; Figure 2E). During the extinction phase, the Scheirer–Ray–Hare test indicated that treatment had a significant effect on CPP scores among the DMSO + saline, DMSO + METH, AHU377 + METH, and valsartan + METH groups (H_(3,108)_ = 21.178, *p* < 0.001). However, neither the effect of testing (H_(3,108)_ = 4.808, *p* > 0.05) nor the interaction between testing and treatment (H_(9,108)_ = 6.768, *p* > 0.05) was significant. Post hoc analysis showed that during test 1, the AHU377 + METH group exhibited significantly higher CPP scores compared to the DMSO + saline group (*p* < 0.05). No significant differences in CPP scores were observed between the DMSO + saline and DMSO + METH groups during Tests 2, 3, and 4 (*p* > 0.05). Across Tests 1 to 4, the CPP scores of the DMSO + METH, AHU377 + METH, and valsartan + METH groups did not differ significantly from those of the DMSO + saline group (*p* > 0.05; Figure 2F).

The Kruskal–Wallis test revealed a significant effect of treatment on CPP scores during the reinstatement phase among the DMSO + saline, DMSO + methamphetamine (METH), AHU377 + METH, and valsartan + METH groups (H = 13.478, *p* < 0.01). Post hoc analysis indicated that the CPP score in the DMSO + METH group was significantly higher than that in the DMSO + saline group (*p* < 0.01). Similarly, the AHU377 + METH group showed significantly elevated CPP scores compared to the DMSO + saline group (*p* < 0.05). However, there were no significant differences among the DMSO + METH, AHU377 + METH, and valsartan + METH groups (*p* > 0.05; Figure 2G).

### 2.3. Experiment 3: The Effect of LCZ696, AHU337 and Valsartan on the Neuronal Plasticity and DA Level in METH CPP Mice

**Figure 3:**
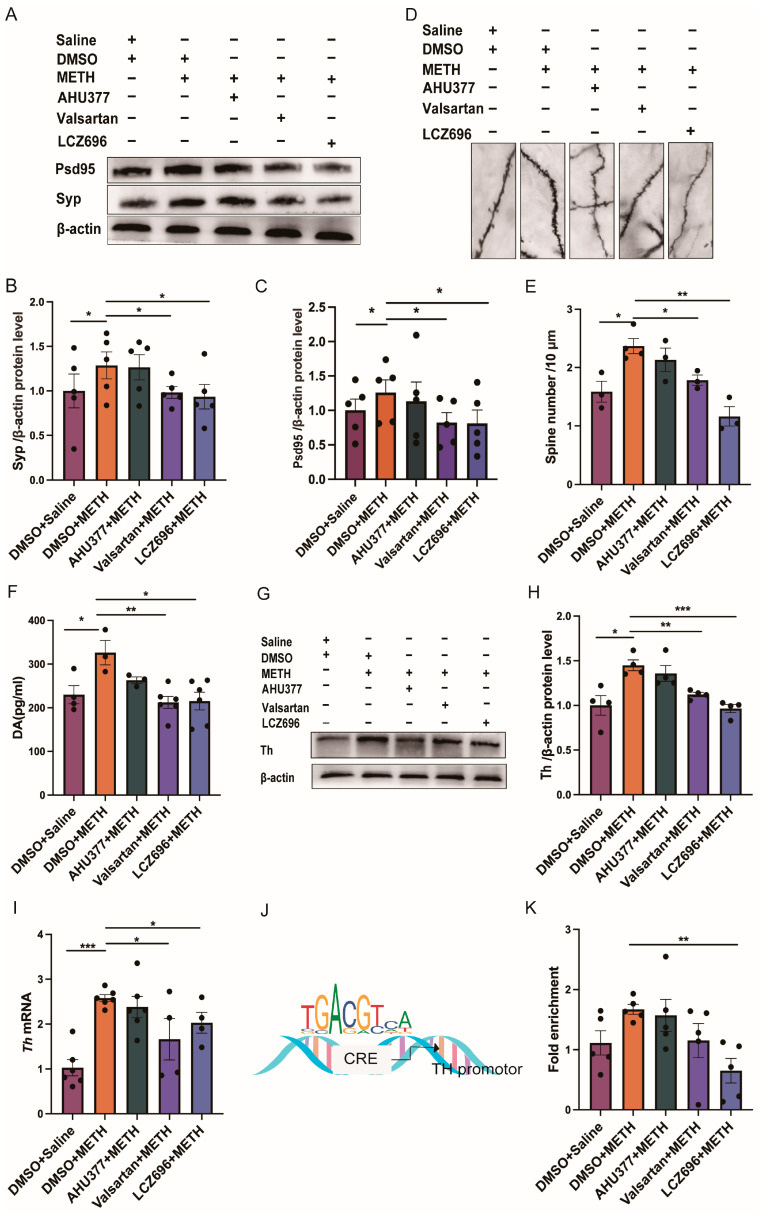
The effect of LCZ696 on synaptic deficits and DA system during the acquisition of METH-induced CPP. (**A**) Representative Western blot bands of Syp and Psd95. (**B**) The protein level of Syp in NAc. (**C**) The protein level of Psd95 in NAc. (**D**) Golgi staining of medium spiny neurons in NAc. (**E**) Analysis of dendritic spine density. (**F**) The level of DA. (**G**) Representative Western blot bands of Th. (**H**) The protein level of Th in VTA. (**I**) The mRNA level of *Th*. (**J**,**K**) The fold enrichment of Creb on *Th* gene. * *p* < 0.05, ** *p* < 0.01, *** *p* < 0.001.

One-way ANOVA revealed that there was no significant group effect on the protein expression of synaptophysin (Syp) and postsynaptic density protein 95 (Psd95) in NAc (Syp: F_(4,20)_ = 1.376, *p* > 0.05; Psd95: F_(4,20)_ = 0.957, *p* > 0.05). Paired *t* test revealed that the protein expressions of Syp and Psd95 were significantly increased in DMSO + METH group, in comparison with DMSO + saline, valsartan + METH, and LCZ696 + METH groups (Syp: DMSO + Saline: *p* < 0.05; valsartan + METH: *p* < 0.05; LCZ696 + METH: *p* < 0.05; Psd95: DMSO + saline: *p* < 0.05; valsartan + METH: *p* < 0.05; LCZ696 + METH: *p* < 0.05). There were no significant differences in the protein expression of Syp and Psd95 between DMSO + METH and AHU377 + METH groups (Syp: *p* > 0.05; Psd95: *p* > 0.05; Figure 3A–C). One-way ANOVA revealed that there was a significant group effect on the density of dendritic spines among all the groups (F_(4,11)_ = 9.51, *p* < 0.01; Figure 3E). Post hoc showed the density of dendritic spines was significantly higher in the DMSO + METH group than in the DMSO + saline, valsartan + METH, and LCZ696 + METH groups (DMSO + saline: *p* < 0.05; Valsartan + METH: *p* < 0.05; LCZ696 + METH: *p* < 0.01). There were no major differences in the density of dendritic spines between the DMSO + METH and AHU377 + METH groups (*p* > 0.05; Figure 3D,E).

One-way ANOVA revealed that there was a major group effect on the DA level in all the groups (F _(4,17)_ = 4.89, *p* < 0.01; Figure 3F). Post hoc indicated that the DA level was significantly higher in the group of DMSO + METH than in the DMSO + saline, valsartan + METH, and LCZ696 + METH groups (DMSO + saline: *p* < 0.05; valsartan + METH: *p* < 0.01; LCZ696 + METH: *p* < 0.05; Figure 3F). The DA level was not significantly different between the DMSO + METH and AHU377 + METH groups (*p* > 0.05; Figure 3F). The Kruskal–Wallis test revealed a significant group effect on Th protein expression levels (H = 16, *p* < 0.01). Post hoc comparisons indicated that the DMSO + METH group exhibited significantly elevated Th protein expression compared to both the DMSO + saline group (*p* < 0.05) and the LCZ696 + METH group (*p* < 0.01). However, no significant difference was observed between the DMSO + METH and AHU377 + METH groups (*p* > 0.05; Figure 3H).

In addition, one-way analysis of variance (ANOVA) demonstrated a significant group effect on *Th* mRNA expression (F_(4,15)_ = 8.9, *p* < 0.001; Figure 3I). Post hoc analysis further revealed that *Th* mRNA levels in the DMSO + METH group were significantly higher than those in the DMSO + saline (*p* < 0.001), valsartan + METH (*p* < 0.05), and LCZ696 + METH groups (*p* < 0.05). Consistent with the protein expression findings, no significant difference in TH mRNA levels was detected between the DMSO + METH and AHU377 + METH groups (*p* > 0.05; Figure 3I).

One-way ANOVA analysis revealed that there was a major group effect on the enrichment of Creb in the *Th* gene promoter (F_(4,20)_ = 3.46, *p* < 0.05; Figure 3K). Post hoc showed the fold enrichment of Creb in *Th* gene promoter was significantly increased in the DMSO + METH group, compared with the LCZ696 + METH group (*p* < 0.01). There were no major differences in the fold enrichment among DMSO + saline, AHU377 + METH and DMSO + METH group (DMSO + saline: *p* > 0.05; AHU377 + METH: *p* > 0.05; valsartan + METH: *p* > 0.05; Figure 3J,K). 

**Figure 4:**
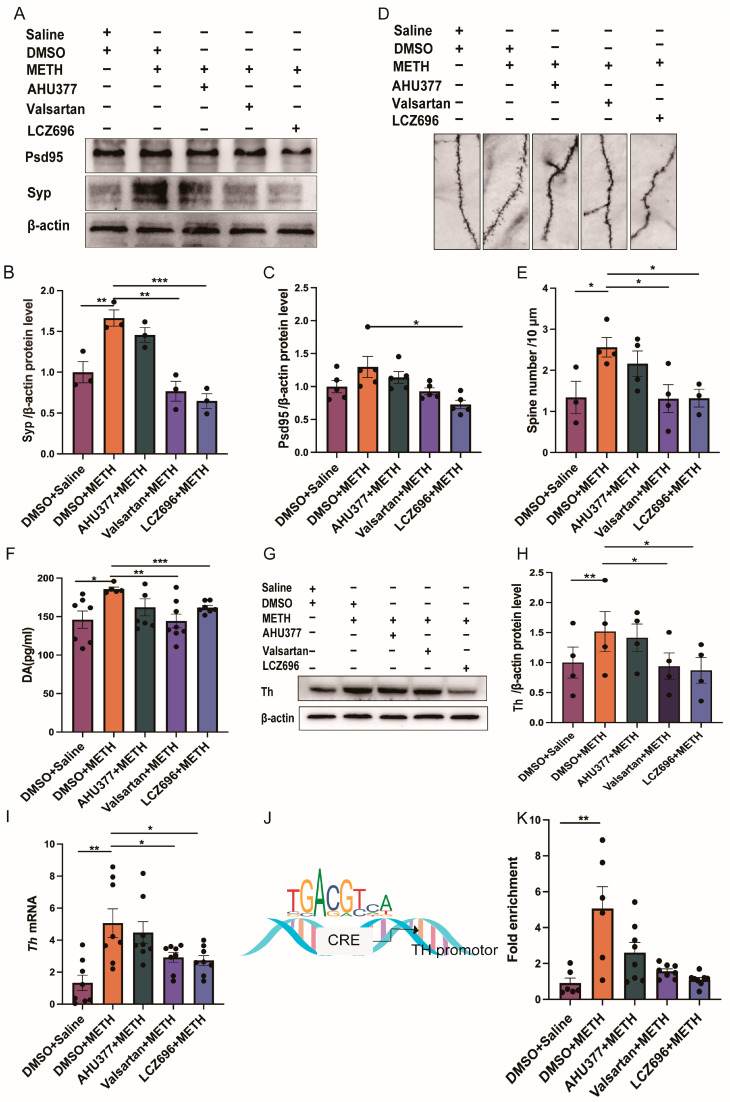
The effect of LCZ696 on synaptic deficits and DA system during METH-induced robust relapse in METH CPP acquisition mice. (**A**) Representative Western blot bands of Syp and Psd95. (**B**) The protein level of Syp in NAc. (**C**) The protein level of Psd95 in NAc. (**D**) Golgi staining of medium spiny neurons in NAc. (**E**) Analysis of dendritic spine density. (**F**) The level of DA. (**G**) Representative Western blot bands of Th. (**H**) The protein level of Th in VTA. (**I**) The mRNA level of *Th*. (**J**,**K**) The fold enrichment of Creb on the *Th* gene. * *p* < 0.05, ** *p* < 0.01, *** *p* < 0.001.

One-way ANOVA revealed significant group effects on the expression of Syp and Psd95 in the NAc (Syp: F_(4,10)_ = 16.68, *p* < 0.001; Psd95: F_(4,20)_ = 4.745, *p* < 0.01). Post hoc comparisons demonstrated that Syp protein expression in the DMSO + METH group was significantly higher than that in the DMSO + saline (*p* < 0.01), valsartan + METH (*p* < 0.01), and LCZ696 + METH groups (*p* < 0.001). Furthermore, Psd95 levels in the DMSO + METH group were significantly higher than those in the LCZ696 + METH group (*p* < 0.05). No significant differences in Syp or Psd95 protein expression were observed between the DMSO + METH and AHU377 + METH groups (Syp: *p* > 0.05; Psd95: *p* > 0.05; Figure 4A–C). One-way ANOVA revealed a significant group effect on dendritic spine density in the treatment groups (F_(4,13)_ = 3.7, *p* < 0.05; Figure 4E). The result showed that dendritic spine density in the DMSO + METH group was significantly higher than that in the DMSO + saline, valsartan + METH, and LCZ696 + METH groups (DMSO + saline: *p* < 0.05; valsartan + METH: *p* < 0.05; LCZ696 + METH: *p* < 0.05). No significant difference in dendritic spine density was observed between the DMSO + METH and AHU377 + METH groups (*p* > 0.05; Figure 4D,E).

One-way ANOVA analysis showed a significant group effect on the DA level in all the groups (F _(4,29)_ = 3.1, *p* < 0.05; Figure 4F). The result showed that the DA level in the DMSO + METH group was significantly higher than that in the DMSO + saline, valsartan + METH, and LCZ696 + METH groups (DMSO + saline: *p* < 0.05; valsartan + METH: *p* < 0.01; LCZ696 + METH: *p* < 0.001). There was no difference in the DA level between the DMSO + METH and AHU377 + METH group (*p* > 0.05; Figure 4F).

One-way ANOVA showed that treatment had no significant main effect on Th protein expression (F_(4,15)_ = 1.346, *p* > 0.05). Paired *t* test indicated that the DMSO + METH group exhibited significantly higher Th protein levels compared to the DMSO + saline, valsartan + METH, and LCZ696 + METH groups (DMSO + saline: *p* < 0.01; valsartan + METH: *p* < 0.05; LCZ696 + METH: *p* < 0.05). No significant difference in Th protein expression was observed between the DMSO + METH and AHU377 + METH groups (*p* > 0.05; Figure 4G,H). The Kruskal–Wallis test revealed a significant main effect of treatment on *Th* mRNA expression (H = 16.2585, *p* < 0.01). The result showed that *Th* mRNA levels in the DMSO + METH group were significantly higher than those in the DMSO + saline, valsartan + METH, and LCZ696 + METH groups (DMSO + saline: *p* < 0.01; valsartan + METH: *p* < 0.05; LCZ696 + METH: *p* < 0.05). There was no significant difference in *Th* mRNA expression between the DMSO + METH and AHU377 + METH groups (*p* > 0.05; Figure 4I). The Kruskal–Wallis test revealed a significant main effect of treatment on Creb enrichment at the *Th* gene promoter across all groups (H = 11.345, *p* < 0.05). Post hoc analysis indicated that Creb enrichment in the DMSO + METH group was significantly higher than in the DMSO + saline group (*p* < 0.01). There were no significant differences in Creb enrichment levels between the DMSO + METH group and the LCZ696 + METH group (*p* = 0.07), AHU377 + METH group, or valsartan + METH group (*p* > 0.05; Figure 4J,K).

### 2.4. Experiment 4: The Effect of Cre Knockout in the Th Gene Promoter of VTA on METH CPP

**Figure 5:**
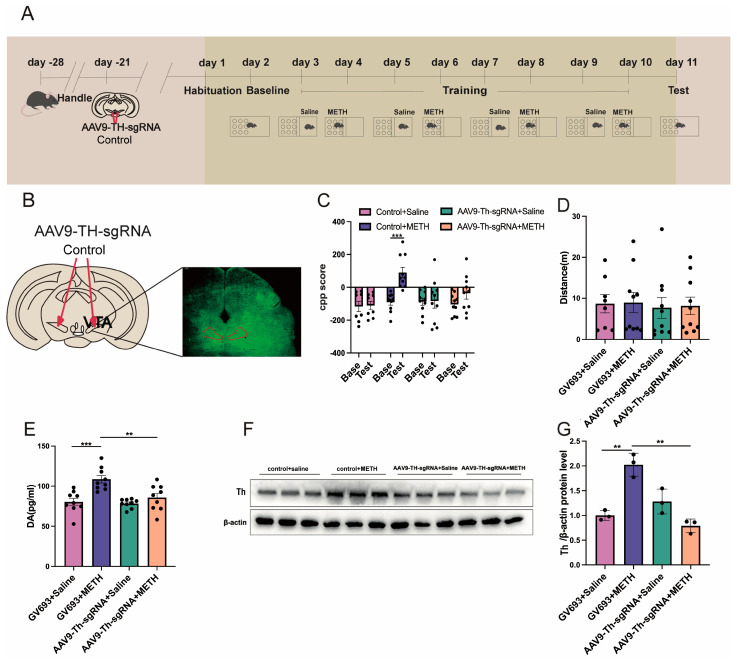
The effect of Cre knockout in VTA Th promoter on METH-associated contextual memory acquisition. (**A**) The experimental timeline. (**B**) The expression of the virus in VTA. The red box indicates the region of viral injection in the VTA. (**C**) The effect of AAV9-Th sgRNA on CPP score in METH mice. (**D**) The locomotor distance of the spontaneous activity test in virus-injected mice. (**E**) The level of DA. (**F**) Representative Western blot bands of Th. (**G**) The protein level of Th in VTA. Group descriptions: GV693 = control viral vector; AAV9-Th-sgRNA = viral vector expressing sgRNA targeting the *Th* gene; saline = vehicle control; METH = methamphetamine exposure during CPP training or priming. ** *p* < 0.01, *** *p* < 0.001.

The expression of the virus in the VTA is shown in Figure 5B. Two-way repeated measures ANOVA revealed significant main effects of the test phase (F_(1,68)_ = 10.537, *p* < 0.01) and treatment group (F_(3,68)_ = 5.268, *p* < 0.01; Figure 5C), as well as a significant test × treatment interaction (F_(3,68)_ = 4.049, *p* < 0.05). Post hoc analysis showed that no significant changes were observed between the Base and test phases in the following groups: control vector + saline (GV693 + Saline), TH-sgRNA vector + saline (AAV9-TH-sgRNA + saline), and Th-sgRNA vector + METH (AAV9-Th-sgRNA + METH) (all *p* > 0.05). In contrast, mice in the control vector + METH (GV693 + METH) group exhibited significantly higher CPP scores during the baseline phase compared to the test phase (*p* < 0.001; Figure 5C). No significant differences in locomotor activity (distance traveled) were found across the four groups (H = 0.8016, *p* > 0.05; Figure 5D).

One-way ANOVA indicated a significant group effect on DA levels (F_(3,32)_ = 10.34, *p* < 0.001; Figure 5E). Post hoc tests revealed that DA levels in the GV693 + METH group were significantly higher than those in the GV693 + saline and AAV9-Th-sgRNA + METH groups (*p* < 0.001 and *p* < 0.01, respectively), whereas no difference was found between the GV693 + saline and AAV9-Th-sgRNA + saline groups (*p* > 0.05).

Similarly, one-way ANOVA showed a significant group effect on Th protein expression (F_(3,8)_ = 24.06, *p* < 0.001). Mice in the GV693 + METH group displayed significantly higher Th expression compared to the GV693 + saline and AAV9-Th-sgRNA + METH groups (*p* < 0.01 for both comparisons). No significant differences were found between the GV693 + saline and AAV9-Th-sgRNA + saline groups (*p* > 0.05; Figure 5F,G). 

### 2.5. Experiment 5: The Effect of Cre Knockout in the Th Gene Promoter of VTA on METH CPP Retrieval

**Figure 6:**
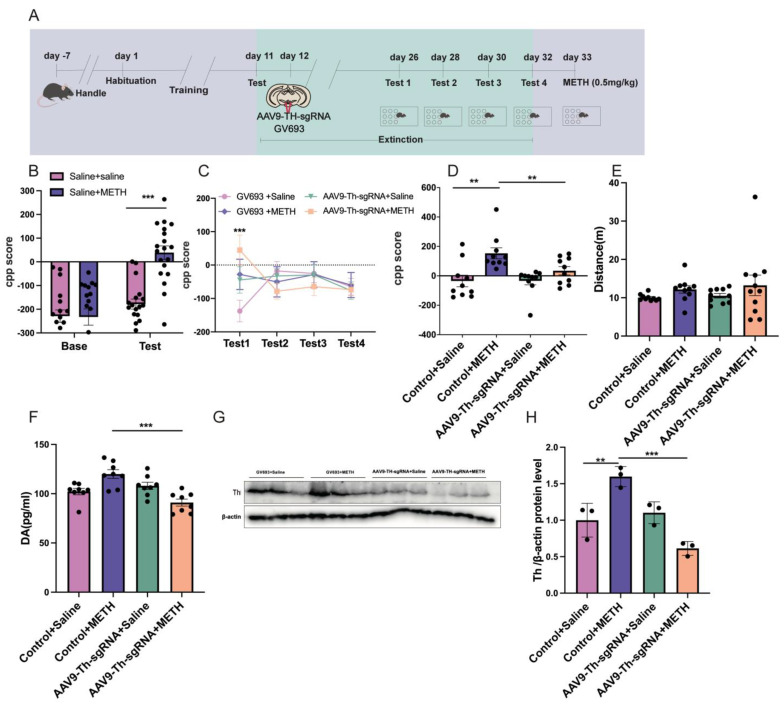
The effect of Cre-mediated knockout in the VTA Th promoter on METH-relapse in mice from the CPP acquisition phase. (**A**) The experimental timeline. (**B**) CPP score of METH-associated contextual memory acquisition mice. (**C**) The effect of AAV9-Th-sgRNA on CPP score in retrieval test mice. (**D**) The effect of AAV9-Th sgRNA on METH-induced robust relapse in METH CPP acquisition mice. (**E**) The locomotor distance of the spontaneous activity test in virus-injected mice. (**F**) The level of DA. (**G**) Representative Western blot bands of Th. (**H**) The protein level of Th in VTA. ** *p* < 0.01, *** *p* < 0.001.

The Scheirer–Ray–Hare test revealed significant main effects of testing, treatment, and their interaction on CPP scores between the saline and methamphetamine groups (testing: H_(1,70)_ = 17.369, *p* < 0.001; treatment: H_(1,70)_ = 9.072, *p* < 0.01; interaction: H_(1,70)_ = 5.952, *p* < 0.05; Figure 6B). Post hoc analysis further indicated no significant difference in CPP scores between the Base and test phases in the saline + saline group (*p* > 0.05). In contrast, the saline + METH group exhibited significantly higher CPP test scores compared to Base (*p* < 0.001; Figure 6B). The Scheirer–Ray–Hare test also revealed significant effects of testing, treatment, and their interaction on CPP scores among the GV693 + saline, GV693 + METH, AAV9-Th-sgRNA + saline, and AAV9-Th-sgRNA + METH groups (test: H_(3,136)_ = 19.294, *p* < 0.001; treatment: H_(3,136)_ = 8.535, *p* < 0.05; interaction: H_(9,136)_ = 17.189, *p* < 0.05; Figure 6C). Subsequent post hoc analysis revealed that during Test 1, the GV693 + saline group showed significantly higher CPP scores compared to the AAV9-Th-sgRNA + METH group (*p* < 0.001). However, no significant differences in CPP scores were observed between these two groups during Tests 2, 3, and 4 (*p* > 0.05). Moreover, during all extinction sessions, no significant differences in CPP scores were found between the GV693 + saline group and the GV693 + METH, AAV9-Th-sgRNA + saline, or AAV9-Th-sgRNA + METH groups (*p* > 0.05; Figure 6C). The Kruskal–Wallis test further confirmed a statistically significant effect of treatment on CPP scores across the GV693 + saline, GV693 + METH, AAV9-Th-sgRNA + saline, and AAV9-Th-sgRNA + METH groups (H = 14.79, *p* < 0.01). Post hoc comparisons indicated that the GV693 + METH group exhibited significantly higher CPP scores than both the GV693 + saline and AAV9-Th-sgRNA + METH groups (GV693 + saline: *p* < 0.01; AAV9-Th-sgRNA + METH: *p* < 0.01; Figure 6D). No significant differences in locomotor activity (measured by total distance traveled) were detected among the groups (H = 5.119, *p* > 0.05; Figure 6E).

The Kruskal–Wallis test revealed a significant difference in DA levels among the groups (H = 15.85, *p* < 0.01). Post hoc comparisons showed that the DA level in the GV693 + METH group was significantly higher than that in the AAV9-Th-sgRNA + METH group (*p* < 0.001). No significant differences in DA levels were observed among the AAV9-Th-sgRNA + saline, GV693 + METH, and GV693 + saline groups (*p* > 0.05; Figure 6F). One-way ANOVA showed that there was a major group effect on the Th protein expression among all the groups (F_(3,8)_ = 18.88, *p* < 0.001). Post hoc analysis revealed that the GV693 + METH group exhibited significantly higher expression of Th protein than both the GV693 + saline and AAV9-Th-sgRNA + METH groups (GV693 + saline: *p* < 0.01; AAV9-Th-sgRNA + METH: *p* < 0.001). There were no significant differences in the Th protein expression between the AAV9-Th-sgRNA + saline and GV693 + saline groups (*p* > 0.05, Figure 6G,H).

### 2.6. Experiment 6: The Effects of LCZ696 on the Other Behaviors of Mice

**Figure 7:**
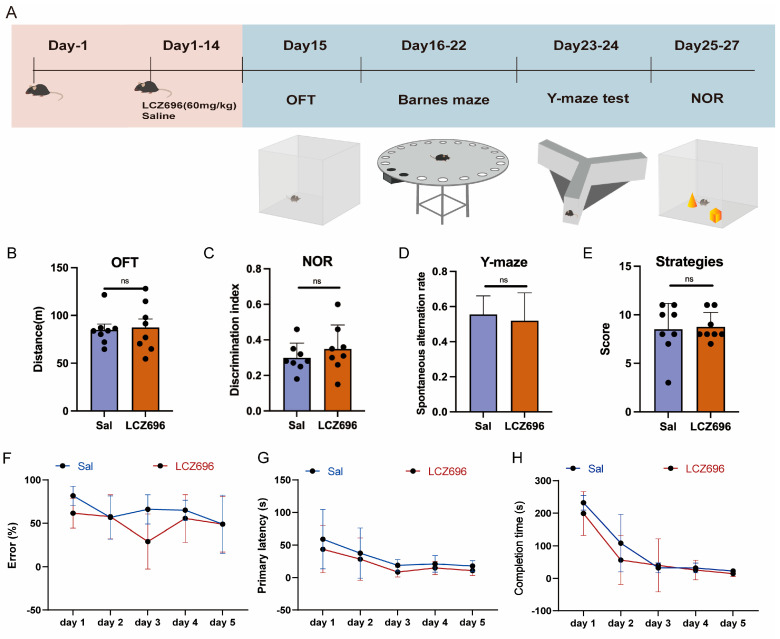
The effects of LCZ696 on mice behavior and cognition. (**A**) The experimental timeline. (**B**) OFT scores in the LCZ696 group and the saline group. (**C**) NOR scores in the LCZ696 group and the saline group. (**D**) Y-maze scores in the LCZ696 group and the saline group. (**E**–**H**) The strategies’ scores, error rate, primary latency, and completion time for the Barnes’ maze. ns: *p* > 0.05.

The independent samples *t*-test showed no significant difference in the distance traveled between the saline group and the LCZ696 group (*p* = 0.827, t = 0.223; Figure 7B). Independent sample *t*-tests revealed that there was no significant difference in the spontaneous alternation rate and the cognitive discrimination index between the saline and LCZ696 group (correct conversion rate: *p* = 0.612, t = 0.516; NOR: *p* = 0.385, t = 0.897; Figure 7C,D). The Wilcoxon rank-sum test indicated no significant difference in Barnes maze strategy scores between the saline and LCZ696 groups (W = 31.5, *p* = 1). Furthermore, the Scheirer–Ray–Hare test revealed no significant differences between these two groups in terms of error rate, primary latency, or completion time (error rate: treatment: H_(1,70)_ = 5.585, *p* < 0.05; day: H_(4,70)_ = 7.429, *p* > 0.05; treatment × day: H_(4,70)_ = 6.263, *p* > 0.05; primary latency: treatment: H_(1,70)_ = 6.124, *p* > 0.05; day: H_(4,70)_ = 15.785, *p* < 0.01; treatment × day: H_(4,70)_ = 1.422, *p* > 0.05; completion time: treatment: H_(1,70)_ = 5.403, *p* < 0.05; day: H_(4,70)_ = 41.52, *p* < 0.01; treatment × day: H_(4,70)_ = 0.626, *p* > 0.05; Figure 7E–H).

## 3. Discussion

The present studies revealed that LCZ696 disrupted the acquisition and retrieval of METH-associated contextual memory, using the animal model of CPP. LCZ696 was also found to inhibit the level of DA by regulating Creb−induced *Th* transcription. The specific Cre knockout in the *Th* gene promoter in the VTA produced similar effects to LCZ696. Further experiments involving the two intermediates, valsartan and AHU377, revealed that valsartan effectively inhibited METH CPP and the retrieval of METH CPP. In contrast, AHU377 demonstrated no significant impact on METH CPP and the retrieval of METH CPP.

To date, this study is the first to investigate the effects of LCZ696 on the rewarding effect of METH. Previous studies have reported the effect of LCZ696 on cognitive functions and emotion. For example, LCZ696 was found to exacerbate cognitive impairment in a colchicine-induced Alzheimer’s disease rat model [22]. Conversely, emerging evidence indicates that LCZ696 may exert neuroprotective effects in drug-induced cognitive deficits: a recent study demonstrated that LCZ696 significantly improved METH-induced cognitive impairment in mice by activating the Nrf2/HO-1 pathway, reducing apoptosis and oxidative stress in the VTA and SH-SY5Y cells [20]. A clinical study including 37 advanced heart failure patients awaiting heart transplants reported that LCZ696 improved depression after a one-year follow-up [23]. Furthermore, clinical studies showed no evidence of the alleged harmful influence of LCZ696 on cognitive function [24,25,26]. In line with this protective potential, the present study also demonstrated that LCZ696 did not affect the cognition and motor behavior of mice. It appears that LCZ696 was effective in reversing the cognitive impairment. The normal cognitive functions remained intact following the treatment with LCZ696.

Several studies have explored the effect of the angiotensin system on drugs with abuse liability. Candesartan, an AT1R blocker, reduced METH self-administration and improved METH-induced cognitive impairment in rats [18,19]. Candesartan has also been found to attenuate METH-induced hyperlocomotion [11,27]. Moreover, the administration of valsartan for 7 consecutive days effectively reduced the induction of morphine tolerance and defecation episodes in naloxone-injected animals [28]. Furthermore, the administration of angiotensin-converting enzyme 1 (ACE1) inhibitors such as captopril or AT1R antagonists like losartan has been shown to significantly reduce locomotor sensitization induced by chronic toluene exposure, potentially through an increased Ang (1–7)/Ang II ratio in the NAc [29]. In addition, ACE inhibition has been reported to enhance endogenous opioid signaling while lowering addiction risk [17]. However, clinical evidence suggests complex dose-dependent interactions: while perindopril (an ACE inhibitor) did not consistently attenuate methamphetamine-induced effects in humans, its selective modulation of diastolic blood pressure and ‘Any Drug Effect’ ratings implies a potential role for RAS in reward processing [12]. These findings align with a randomized double-blind clinical trial demonstrating that losartan (an AT1R antagonist) modulated behavioral responses to social punishment and reward by altering reaction times and arousal levels [30]. Building on these findings, the present study showed that LCZ696 significantly attenuated METH-induced reward behavior in a CPP model, suggesting that RAS modulation may influence both social and drug-related reward processing. Collectively, these clinical observations establish RAS as a key regulator of reward circuitry. Building on this foundation, the present study showed that LCZ696—a dual RAS modulator targeting both AT1R and NEP—significantly attenuated METH-induced reward behavior in a CPP model. This suggests that RAS modulation may exert broad control over reward processing, spanning both social (as in [20]) and drug-related domains.

The results of this study further explore the underlying mechanism of the inhibitory effect of LCZ696 on METH CPP. Overexpression of the Th in DA neurons has been shown to increase sensitivity to METH [31]. A previous study reported that toll-like receptor 4 (TLR4) in dopaminergic neurons induces Th expression through Creb, which may modulate impulsivity and be associated with the onset of alcohol drinking [32]. Similar mechanisms have been observed with other psychostimulants. For example, Creb-induced upregulation of Th expression has been implicated in the formation of cocaine-associated CPP behavior [33]. It appears that the regulation of Th expression by METH-induced Creb could play a crucial role in the DA level, which is associated with the rewarding effect of METH. In the present study, METH resulted in increased binding between Creb and CRE in the *Th* gene promoter, thereby enhancing the synthesis of Th and DA. Furthermore, the AT1R inhibitor valsartan in LCZ696 effectively reduced the abnormally increased binding of Creb and CRE in the *Th* gene caused by METH.

Since LCZ696 was composed of two chemicals, valsartan and AHU377, it produced dual effects that block both AT1R and NEP. Additional studies were carried out to examine the separate effects of these two compounds on METH CPP and DA level. The present studies demonstrated that valsartan, rather than AHU377, mainly contributed to the effect of LCZ696 on METH CPP. Several studies reported that NEP is related to cognitive impairment and may predispose individuals to neurodegenerative diseases such as Alzheimer’s disease [34]. NEP knockout mice have exhibited significant cognitive impairments in both working memory and spatial memory. NEP inhibitors can extend the duration of enkephalin effects, thereby promoting the analgesic, sedative, and antidepressant effects [35]. Another study revealed that LCZ696 could improve cognitive impairment induced by METH [20] Enkephalins could block the reinforcing effect of sucrose administration but were found to be ineffective against chronic morphine administration [36]. In the present study, AHU377 showed no significant effect on attenuating the effects induced by METH on CPP. Therefore, different mechanisms may mediate the effects of AHU377 in neurodegenerative disorders as opposed to the rewarding effect of METH.

Nevertheless, the limitations of this study should be acknowledged. Firstly, only male mice were used in the present study. The effects of fentanyl on the contextual reward, using the CPP paradigm, in female and male rats showed that fentanyl produced significant effects in both sexes but was more potent in males [37]. For example, the GluN2B-selective antagonist Ro 63-1908 inhibited the acquisition of METH CPP in male rats but only attenuated CPP in female rats. Whether LCZ696 produces similar effects on female mice remains unknown [38]. Secondly, only the CPP model was used to examine the effects of LCZ696 on METH, which limits our understanding of its broader behavioral impact. The CPP paradigm primarily reflects interoceptive reward-related cues, while self-administration paradigms better model motivational aspects of drug-seeking behavior [39]. Future studies using METH self-administration models could help elucidate the reinforcing and motivational effects of LCZ696.

In summary, LCZ696 disrupted the acquisition and retrieval of METH-associated contextual memory via the modulation of Creb-induced Th transcription. AT1R inhibition may be an important target for the treatment of METH use disorder.

## 4. Materials and Methods

### 4.1. Animals

Male C57BL/6J mice (18–20 g; Zhejiang Academy of Medical Sciences, Hangzhou, China) were kept in a room with a standard environment (temperature of 22 to 24 °C and humidity of 40 to 70%). Mice were housed in groups and were allowed free access to food and water. Behavioral testing was performed during the dark phase with a 12 h light–dark cycle. This study was conducted according to the guidelines of the Institutional Animal Care and Use of Ningbo University. In total, 200 male C57BL/6J mice were used across all experiments.

### 4.2. Drugs

METH (>90% purity) was obtained from the Ningbo Public Security Bureau and was dissolved in 0.9% saline. The use of methamphetamine in this study was approved by the Ningbo Public Security Bureau. LCZ696, AHU377, and valsartan were purchased from Shanghai Ruihui Chemical Co., Ltd., Shanghai, China. LCZ696 (60 mg/kg), valsartan (30 mg/kg), and AHU377 (30 mg/kg) [20] were dissolved in dimethyl sulfoxide (DMSO) (Sinopharm Chemical Reagent Co., Ltd, Shanghai, China)as a stock solution and used at a final concentration of 10% DMSO and 90% saline. The AAV9-Th-sgRNA and GV693 virus were purchased from GeneChem (Shanghai, China). METH was administered via intraperitoneal injection, and the virus was administered by stereotactic injection. Other drugs were administered by oral gavage.

### 4.3. Conditioned Place Preference (CPP)

The CPP (AniLab Scientific Instruments Co., Ltd., Ningbo, China) apparatus consisted of three compartments (15 × 15 × 37 cm). Two conditioning compartments were separated by a smaller central compartment, which was distinguished by floor textures and wall colors. The subjects were placed in the experimental environment for 3 days before the CPP training. During the baseline phase, mice were placed in the middle chamber and allowed to freely explore the compartments for 15 min. The preferred side was selected as the saline-paired chamber, and the other side was set as the METH-paired chamber. In our study, we employed a biased CPP protocol, wherein the drug was consistently paired with the environment that mice initially found least preferable during the pretest phase. This approach is designed to mitigate the ceiling effect that can occur when a drug is paired with a naturally preferred environment, thereby enhancing the sensitivity of the CPP paradigm to detect the rewarding effects of the drug. The biased CPP protocol is widely utilized in addiction research and has been demonstrated to be effective in various studies [40,41]. On the odd days of the conditioning phase, mice were injected with saline and placed into the saline-paired chamber for 30 min. On the even days, mice were injected with METH (2.5 mg/kg). We selected the 2.5 mg/kg dose because previous studies have demonstrated its effectiveness in eliciting the rewarding effects of METH [2,42]. The animals were confined to the METH-paired chamber for 30 min. During the testing phase, mice performed the place preference test, which was identical to the baseline test. The CPP scores were expressed as the time spent in the METH-paired chamber minus the time spent in the saline-paired chamber. After each trial, the apparatus was cleaned with 75% ethanol to eliminate any residual odor. Extinction was carried out in two stages. During the first and second weeks, the animals were housed individually in home cages. During the third week, mice were placed in the middle chamber and allowed to freely explore the compartments for 15 min. Extinction testing was carried out, following the stage of extinction. Following an intraperitoneal injection of METH (0.5 mg/kg), each animal was placed into the middle chamber and allowed to freely explore the compartments for 15 min. The time spent in different compartments was recorded. This design was chosen to align with viral intervention timelines and prioritize isolation of memory destabilization mechanisms, consistent with protocols demonstrating that prolonged abstinence in a home-cage environment reduces drug-associated memory maintenance and facilitates extinction [43]. The CPP score was calculated as the time spent in the METH-paired chamber minus the time spent in the saline-paired chamber during the post-conditioning test phase.

### 4.4. Locomotor Activity

The locomotor activity test was conducted to evaluate general motor function. The animals were placed individually inside white plastic cages (40 × 40 × 40 cm) and allowed to freely explore for 15 min. The total distance traveled during the last 10 min was recorded using a behavioral analysis system (Anilab Ltd., Ningbo, China). This test was conducted on a separate day prior to the open field test to avoid behavioral interference. After each session, the apparatus was cleaned with 75% ethanol to remove any residual odor.

### 4.5. Open Field Test (OFT)

The open field test was used to assess both locomotor activity and anxiety-like behavior. The apparatus consisted of five white acrylic panels forming a 40 × 40 × 40 cm open box. The test was divided into two phases. On the first day (adaptation phase), animals were allowed to freely explore the open field for 15 min. On the second day (testing phase), each mouse was again placed in the center of the open field and allowed to explore for 15 min. Both the total distance traveled and the time spent in the central zone during the last 10 min on day two were recorded and analyzed. These two parameters respectively reflected general activity and anxiety-like behavior. The apparatus was thoroughly cleaned with 75% ethanol after each trial.

### 4.6. Barnes Maze

The Barnes maze was made of a white circular platform with a diameter of 122 cm. The platform had 18 equidistant round holes with a diameter of 5 cm around the edge of the platform. There was a small, dark escape box placed below one of the holes. The other seventeen holes were empty without any boxes. The animals escaped to the target box through the target hole. The experiment was divided into three stages: adaptation (1st day), training (2nd–6th day), and testing (7th day). The latency to reach any hole, the latency to reach the target box, and the number of errors made by each animal were recorded. One error was defined as the animal putting its head into or exploring any non-target hole, including focusing on exploring the same non-target hole. Finally, the strategy score was calculated as the average of the three scores mentioned above.

### 4.7. Y-Maze Test

The Y-maze test consisted of three identical long arms (50 × 18 × 35 cm, 120°). The Y-maze test included an adaptation and a testing period. On the first day, one of the long arms was closed, and mice were allowed to freely explore the other two arms for 10 min. On the second day, the test was conducted by placing the mice in the middle of the Y-maze. The mice freely explored for 10 min, with a camera recording the number of times the mice consecutively explored the three long arms and the total number of entries into the arms during the last 5 min. The standard for a mouse to enter each arm was that all four limbs were completely inside. Entering three different arms in sequence (e.g., ACB, BAC, CBA, etc.) was considered an alternation. The main measure was the spontaneous alternation rate of the mice. It was calculated as follows: %Alternation = (Number of successful alternations/Total arm entries − 2) × 100%.

### 4.8. New Object Recognition (NOR)

The novel object recognition (NOR) test consisted of three phases: adaptation, training, and testing. On the first day (adaptation phase), mice were placed individually in the empty testing arena and allowed to freely explore for 10 min. During the training phase (Day 2), two identical objects were placed in the arena, and mice were again allowed to explore for 10 min. In the testing phase (Day 3), one of the familiar objects (a 5 cm × 7.5 cm cone) was replaced with a novel object (a 5 cm × 5 cm × 5 cm cube), and the animals were allowed to explore for another 10 min. A 24 h interval was maintained between each phase. After each trial, the surface of the arena and the objects were thoroughly cleaned with 75% ethanol to eliminate residual odors.

Object recognition was defined as the animal’s nose and whiskers approaching within 2 cm of the object while the body was oriented toward it and displaying active movement. Brief incidental contact with the body alone was not considered valid exploration. The discrimination index (DI) was calculated using the following formula:

DI = (Time exploring novel object − Time exploring familiar object)/(Total exploration time) × 100%.

### 4.9. Viral Injection

After anesthesia, mice were fixed on a stereotaxic apparatus. AAV9-Th-sgRNA (6.03 × 10^12^ copies/mL) or GV693 (an AAV9-based control vector carrying a non-targeting sgRNA sequence [CGCTTCCGCGGCCCGTTCA] under the U6 promoter) was bilaterally injected into the VTA (AP: −3.2 mm; ML: ±0.5 mm; DV: −4.5 mm) at 0.1 μL/min, 0.5 μL per side. The needle was retained for 10 min. Mice were randomly assigned to the Th-sgRNA or GV693 group. GV693 served as a negative control to account for potential off-target or viral effects. All surgical parameters and handling procedures were kept identical between groups. Following injection, mice were allowed to recover for 3 weeks to ensure stable viral expression.

### 4.10. Quantitative Real-Time PCR

Total RNA was extracted from the VTA of mouse brain tissues using TRIzol reagent. The RNA was diluted to 1000 ng with DEPC water and transcribed with TransScript All-in-One First-Strand cDNA Synthesis SuperMix (AT341, TransGen Biotech, Beijing, China). The cDNA was quantified by quantitative reverse transcription-PCR (qRT-PCR) using trans-Start Tip Green qPCR SuperMix (AQ141, TransGen Biotech, Beijing, China). Each reaction was run three times and analyzed using the 2^−ΔΔCt^ method. The primers were designed by the Beijing Genomics Institution (Shenzhen, China). (*Th* primers: Forward: GAGGTATACGCCACGCTGAA; Reverse: GGAAGCCAGTCCGTTCCTTC)

### 4.11. Western Blotting

Total RNA was extracted from the VTA of mouse brain tissues using TRIzol reagent. The samples were lysed with the radioimmunoprecipitation assay (RIPA) lysis buffer (New Cell & Molecular Biotech Co., Ltd., Gujarat, India) and Phenylmethanesulfonyl fluoride (PMSF) (Beijing Solarbio Science & Technology Co., Ltd., Beijing, China) (100:1). Protein samples were collected following centrifugation at 13,000 rpm at 4 °C, and the protein levels were quantified with the Pierce BCA protein assay kit (Beyotime Biotechnology, Shanghai, China). The protein was diluted to the same concentration with phosphate-buffered saline (PBS) and 5 × SDS-PAGE loading buffer (Beyotime Biotechnology, Shanghai, China). The final concentration to load the running gel was 1.5 μg/μL (total 30 μg to load). Protein samples were separated by 10% SDS-PAGE based on their different molecular weights and transferred to PVDF membranes (Bio-Rad, Hercules, CA, USA). Blocking was performed with 5% non-fat dried milk for 2 h, followed by incubation of the primary antibodies overnight at 4 °C and the corresponding secondary antibodies for 2 h at room temperature. The following antibodies were used: anti-beta Actin (β-actin, 1:1000; ab8226, Abcam, Cambridge, UK), anti-TH antibody (TH, 1:1000; A5079, ABclonal, Woburn, MA, USA), anti-PSD95 antibody (PSD95, 1:3000; 20665-1-AP, Proteintech, Rosemont, IL, USA), and anti-SYP antibody (SYP, 1:2000; sc-17750, Santa Cruz Biotechnology). The membrane was exposed to an ECL chemiluminescence reagent (Advansta, Menlo Park, CA, USA), and the Western blot images were observed on a Chemi Scope 6000 Touch (Qinxiang Scientific Instrument, Shanghai, China) and analyzed using Image J.

### 4.12. Enzyme-Linked Immunosorbent Assay

Dopamine levels were measured in homogenates of VTA tissues. The ELISA kit was applied for this experiment (Qiaodu Biological Technology Co., Ltd., Shanghai, China). Saline was added to the samples for homogenization, and the samples were centrifuged at 3000 rpm for 10 min to obtain the supernatant. Then, 50 μL standards with different concentrations were added to the standard wells. Other wells were spiked with 40 μL of the sample and 10 μL of the sample diluent. Subsequently, 100 μL of horseradish peroxidase-labeled detection antibodies was added to the standard wells and sample wells and incubated at 37 °C for 60 min. Finally, 50 μL of reaction substrates A and B were added to all the wells and incubated for 15 min at 37 °C. After the incubation was completed, 50 μL of stop solution was added, and the absorbance was measured at a wavelength of 450 nm. A standard curve was drawn, and the concentration of the object to be detected in the sample was determined.

### 4.13. Chromatin Immunoprecipitation (CHIP)

Fresh VTA tissues were collected and immediately fixed in 1% formaldehyde (diluted from 16% stock; Cell Signaling Technology, Danvers, MA, USA) at room temperature for 10 min to crosslink DNA–protein complexes, followed by quenching with 125 mM glycine for 5 min. The tissues were then homogenized and lysed in ChIP lysis buffer, and chromatin was fragmented by enzymatic digestion combined with ultrasonic shearing to achieve DNA fragments of 200–1000 bp. After centrifugation, the supernatant containing soluble chromatin was incubated overnight at 4 °C with anti-CREB antibody (CREB, 1:50; 9197, Cell Signaling Technology), while normal rabbit IgG served as a negative control. Immune complexes were captured using protein A/G agarose beads, followed by sequential washes with low-salt, high-salt, LiCl, and TE buffers to remove nonspecific binding. Bound complexes were eluted and subjected to reverse crosslinking at 65 °C overnight, followed by proteinase K treatment. DNA was purified using a spin column-based DNA purification kit (e.g., Qiagen, Düsseldorf, Germany), and enrichment of Creb binding to the Th promoter was assessed by quantitative PCR using specific primers (Forward: 5′-TCCGTCGCCCTCGCTCTGTGCCCACC-3′; Reverse: 5′-CTGGTGGTCCCGAGTTCTGTCACCAC-3′).

### 4.14. Golgi-Cox

The solutions A and B from the kit (Hito Golgi-Gox OptimStainTM PreKit, manufactured by Hitobiotec Corp., Kingsport, TN, USA, and purchased from Shanghai Maokang Biotechnology Co., Ltd., Shanghai, China) were mixed evenly 24 h in advance. The NAc tissue samples were collected and rinsed with distilled water. Then, the samples were placed in the mixture of A and B and stored at room temperature in the dark for 24 h. The soaking solution was changed, and the samples were stored at room temperature in the dark for an additional 2 weeks. The tissues were transferred to solution C, immersed in the dark for 12 h at 4 °C, and replaced with new solution C. The tissues were further stored in the dark for 48 h at 4 °C. The samples were prepared into sections of 100 μm thickness by a vibrating microtome and were transferred to gelatin-coated microscope slides containing solution C, and dried at room temperature for 24 h. The slides were rinsed twice with distilled water for 3 min each time. A mixture of Solution D, Solution E, and water at a 1:1:3 ratio was prepared, and the slides were placed in the mixture for 10 min. The slides were rinsed with distilled water twice for 4 min each time, and the sections were dehydrated in 50%, 75%, and 95% ethanol for 5 min at each concentration gradient. The sections were then dehydrated in absolute ethanol four times for 5 min each time. Subsequently, the sections were made transparent in xylene twice for 5 min each time. The samples were sealed, and images were acquired under a microscope. Spine density was calculated manually from 3–5 secondary dendrites per neuron, averaged across 4–6 neurons per mouse, using ImageJ software 2.14.0.

## Data Availability

The data that support the findings of this study are available from the corresponding authors upon reasonable request.
